# Triple Reuptake Inhibitors: The Next Generation of Antidepressants

**DOI:** 10.2174/157015908787386078

**Published:** 2008-12

**Authors:** David M Marks, Chi-Un Pae, Ashwin A Patkar

**Affiliations:** 1 Department of Psychiatry, Duke University Medical Center, Durham, North Carolina, USA; 2 Department of Psychiatry, The Catholic University of Korea College of Medicine, Seoul, South Korea

## Abstract

Depression has been associated with impaired neurotransmission of serotonergic, norepinephrinergic, and dopaminergic pathways, although most pharmacologic treatment strategies for depression enhance only serotonin and norepinephrine neurotransmission. Current drug development efforts are aimed at a new class of antidepressants which inhibit the reuptake of all three neurotransmitters in the hope of creating medications with broader efficacy and/or quicker onset of action. The current review explores limitations of presently available antidepressants and the history and premise behind the movement to devise triple reuptake inhibitors. The evidence for and against the claim that broader spectrum agents are more efficacious is discussed. Examples of triple reuptake inhibitors in development are compared, and preclinical and clinical research with these agents to date is described.

## INTRODUCTION

Since the catecholamine hypothesis of depression was first described in the 1960’s, most antidepressant drug development has targeted the enhancement of monoamine neurotransmission. For decades tricyclic antidepressants (TCAs), which inhibit the reuptake of norepinephrine and serotonin, were the principal treatment choice for clinicians; unfortunately these agents have classic side effects as a result of histaminergic, cholinergic, and alpha-adrenergic receptor antagonism. Additionally, TCAs have a low therapeutic index related to quinidine-like cardiac conduction effects which make them quite dangerous in overdose. Monoamine oxidase inhibitors have also been in use for approximately fifty years, but their inhibition of monoamine catabolism predisposes them to drug-drug interactions as well as interactions with dietary tyramine. In the past two decades, antidepressant drug development efforts have focused on improving tolerability which has led to molecules that specifically inhibit serotonin reuptake (SSRI) or both serotonin and norepinephrine reuptake (SNRI). These agents have more benign side effect profiles than TCAs or monoamine oxidase inhibitors (MAOIs), although they have not shown advantages in efficacy or in onset of antidepressant response [41,48]. To date, only 65% of patients treated with antidepressants experience therapeutic response [41,48,36,39], even after multiple steps of antidepressant treatment, augmentation, and switching as noted in the recent Sequenced Treatment Alternatives to Relieve Depression (STAR-D) trial [38]. Additionally, typical onset of action of antidepressants does not occur until approximately 2-4 weeks [33,47]. Current development efforts include the evaluation of triple reuptake inhibitors which block the reuptake of serotonin, norepinephrine, and dopamine from the synapse. It is theorized that the additive effect of enhancing neurotransmission in all three monoamine systems (“broad spectrum”) may lead to improved efficacy and quicker onset of antidepressant response. 

## THE TRIPLE-ACTION HYPOTHESIS

Copious evidence links depression to deficiencies in neurotransmission of the monoamines serotonin, norepinephrine, and dopamine [7,34,35,43,54]. As described, TCAs and MAOIs became used widely for depression after they were serendipitously discovered to be efficacious in depressed patients. Subsequent study demonstrated that these medications work by inhibiting the norepinephrine and serotonin transporters (e.g. TCAs) [2] and by inhibiting the intracellular catabolism of norepinephrine and serotonin (e.g. MAOIs). Simultaneously, depletion studies revealed that depression was a consequence of deficient norepinephrine and serotonin [8,9,55]. Rational drug design later led to SSRIs and SNRIs which have successfully led to reduced side effect burden as a result of their selectivity for monoamine reuptake sites. Other antidepressants have been developed which enhance norepinephrine and serotonin neurotransmission *via* other mechanisms; such medications include mirtazepine (presynaptic alpha-2 adrenergic antagonist), as well as trazodone and nefazodone (primarily presynaptic and postsynaptic 5-HT2 antagonists). Less attention has been given to affecting dopamine transmission in depression, although data indicate the important role of mesolimbic dopamine in moderating motivation and reward-related behavior which are typically disrupted in depression [29,44]. Furthermore, antidepressants have been shown to sensitize mesolimbic dopamine receptors in animal and human studies, findings which have led to the hypothesis that enhancing synaptic dopamine availability may lead to more rapid antidepressant response [44]. The dopamine and norepinephrine reuptake inhibitor bupropion was developed in the 1980’s as an antidepressant [53], and it has since been repeatedly shown to boost the therapeutic response to norepinephrinergic and/or serotonergic antidepressants (and decrease sexual side effects) when used as augmentation [5,12,56]. Additional data indicate that the stimulant class of medications, which induce release and block reuptake of dopamine and norepinephrine, augment and hasten antidepressant response when combined with TCAs [10,18,52], MAOIs [13,14], and SSRIs/SRNIs [27, 49]. Finally, dopamine agonists themselves (bromocriptine, pergolide) have shown efficacy as augmenting agents with antidepressants in open label studies [20,21 cited in 44]. Thus, it seems that serotonin, norepinephrine, and dopamine systems are all related to the pathophysiology of depression and as such are relevant targets for pharmacological intervention. This premise has ushered the development of medications which enhance neurotransmission of all three systems in an effort to provide more reliable efficacy and quicker therapeutic effect. 

## IS BROADER BETTER?

Prior to the 1980’s, drug candidates were identified by testing laboratory-derived compounds in animal models and observing the effects. As biochemical and genetic research identified the molecular mechanisms that underlie disease, drug development began to focus on increasing selectivity with the goal of affecting only the target molecule identified as relevant in order to minimize side effects [16]. However, clinical studies of complex disorders as diverse as cancer and schizophrenia reveal that “dirty” drugs affecting a variety of targets tend to have better efficacy [16].

In a reversal of the move towards selectivity that led to the SSRIs in the 1980’s and 1990’s, the SNRIs were developed under the premise that a broader spectrum of neurotransmitter reuptake inhibition would lead to greater antidepressant efficacy and/or more rapid onset of action. Data accumulated to date is mixed regarding the usefulness of this strategy. With regard to rapidity of onset, venlafaxine performed well in a placebo-controlled trial in hospitalized depressed patients, demonstrating efficacy benefit within one week of treatment initiation [17]. Similarly, venlafaxine produced earlier time to remission of depression compared to SSRIs in an open-label trial [40]. A meta-analysis of eight studies comparing venlafaxine to SSRIs and placebo demonstrates earlier time to remission in venlafaxine-treated patients consistently across age and gender groups [11]. The authors also cite remission rates of 45%, 35%, and 25% in patients treated with venlafaxine, SSRIs, and placebo respectively, yielding an odds ratio for remission of 1.5 favoring venlafaxine over SSRIs [50]. Two more recent large meta-analyses of studies comparing SNRIs to SSRIs revealed a smaller efficacy advantage in favor of SNRIs (4.3-5.9% higher remission rate) [30,32]. In these later meta-analyses, the number needed to treat (NNT) statistic shows that 17-24 patients would need to be treated with SNRIs to yield one additional responder [30,32], confronting the notion that SNRIs offer a clinically relevant advantage over SSRIs with respect to likelihood of achieving remission of depression.

Other data endorse the thrust towards increased neurotransmitter selectivity. In particular, a meta-analysis of the highly selective SSRI escitalopram (S-isomer of citalopram) suggests that this medication is superior in efficacy to other SSRIs and to the SNRI venlafaxine (grouped together) on the outcomes of response rate, remission rate, and overall treatment outcome [24]. A second meta-analysis indicates more rapid onset of action of escitalopram compared to other SSRIs and venlafaxine extended-release (grouped together) [22]. Thus, it remains controversial whether certain antidepressants confer clinically relevant advantages in rapidity of onset or overall efficacy for depression, and whether such differences are related to the breadth of their neurotransmitter reuptake inhibition. 

It should also be noted that currently available dual reuptake inhibitors differ in their relative potencies at monoamine transporters. Milnacipran blocks serotonin and norepinephrine reuptake equally, whereas greater selectivity at serotonin reuptake sites is characteristic of venlafaxine (30-fold) and duloxetine (10-fold) [37]. Clinical ramifications of these *in vitro* differences in selectivity are poorly understood.

## EXAMPLES OF COMPOUNDS IN DEVELOPMENT

Despite the structural similarity of the norepinephrine, serotonin, and dopamine transporters, synthesis of bioavailable and safe molecules which appreciably inhibit all three transporters has been challenging [46]. Additionally, the optimal selectivity at the three transporter sites is unknown, and it is plausible that different potency ratios mean different clinical effects. Two families of compounds in development are analogs of the dual reuptake inhibitors milnacipran and venlafaxine. In particular, racemic analogs of venlafaxine referred to as PRC025 and PRC050 are highly potent at human norepinephrine (NE), serotonin (SER), and dopamine (DA) transporters and inhibit the reuptake of these monoamines into rat brain synaptosomes [39]. These compounds exhibited antidepressant-like characteristics equal to imipramine in well-accepted rat models of antidepressant effect; both PRC025 and PRC050 increased time spent swimming and reduced time spent immobile in the forced swim test and reduced time spent immobile in the tail suspension test [39]. Several milnacipran derivatives have been developed in search of molecules with more potent N-methyl-D-aspartic acid (NMDA) antagonism [23,42 cited in 37]. More recently, analogs have been synthesized to evaluate their relative monoamine transporter inhibition potency and selectivity. An isomer of one such analog (-)-8h functions as a triple reuptake inhibitor *in vitro* [37]. To date, animal or human antidepressant studies have not been published with this compound.

DOV Pharmaceutical, Inc. has developed triple reuptake inhibitors from a class of azabicyclohexanes chemically related to bicifadine. Three of these compounds (DOV 216,303, DOV 21,947, and DOV 102,677) have been shown to block transport of human recombinant NE, SER, and DA transporters with clinically-relevant potency (Table **1**) [4,34,44, 45]. Also, all three of these compounds demonstrated antidepressant properties in rodent models; 21,947 reduced immobility during forced swim test and tail suspension test [45], 102,677 reduced immobility during forced swim test [34], and 216,303 reduced immobility during forced swim test and reversed tetrabenazine-induced ptosis [44]. Human studies with DOV 216,303 show that it is well-tolerated at clinically appropriate doses with minor gastrointestinal side effects ranging from 19-57% [4,44,]. A small citalopram-controlled clinical trial of DOV 216,303 (N=67) yielded significant improvements in Hamilton Depression Rating Scale (HAMD) scores in both groups at both the one-week and two-week time points, although the study lacked a placebo group [44]. Bicifadine (1-p-tolyl-3-azabicyclo[3.1.0]hexane) itself has been pharmacologically characterized, and it has been shown to inhibit monoamine neurotransmitter uptake by recombinant human transporters *in vitro* with a relative potency of NE:SER:DA of 1:2:17 [3]. To date, published preclinical research has focused on the potential antinociceptive properties of bicifadine [3], although its utility as an antidepressant warrants exploration.

The novel triple reuptake inhibitor tesofensine (NS 2330) has not been systematically studied regarding its clinical or preclinical antidepressant effects. Similar to antidepressants [6], this agent has demonstrated neuroprotective effects including increasing brain derived neurotrophic factor (BDNF) and neuronal proliferation in the rat hippocampus [26].

It is likely that other triple reuptake inhibitors are in various developmental phases, and the current discussion of compounds in development should not be considered exhaustive. A summary of described compounds appears below in Table **1**.

## OTHER POTENTIAL INDICATIONS FOR TRIPLE REUPTAKE INHIBITORS

Like other classes of antidepressant medications, triple reuptake inhibitors likely hold promise for a variety of therapeutic indications. One emerging area of research concerns the potential antinociceptive effects of triple inhibitors, which is expected given the copious data supporting the utility of TCAs and SNRIs for pain syndromes. Preclinical research with bicifadine demonstrates its antinociceptive effects in animal models of acute, persistent, and chronic pain including inflammatory, visceral, and nociceptive paradigms. These effects were reduced in some experimental conditions by the coadminstration of sulpride (a dopamine-2 receptor antagonist), suggesting that enhancement of dopamine neurotransmission is important for the full antinociceptive effect of bicifadine [3].

The prodopaminergic potential of tesofensine led to a proof-of-concept study of this agent in the treatment of Parkinson’s disease (PD). In this adequately-powered study (N=261) with multiple dosage arms corresponding to up to 77% DA transporter occupancy, tesofensine did not outperform placebo [19]. Two smaller open-label studies of tesofensine and the related compound brasofensine also failed to demonstrate benefit in PD [15,51]. One possible explanation is homeostatic reduction in DA synthesis and release [19]. In contrast, in a phase IIa pilot study in Alzheimer’s disease, tesofensine treatment was associated with cognitive improvements [51 cited in 19]; the physiological mechanism of this observation is unclear, although it has been proposed that tesofensine indirectly stimulates cholinergic neurotransmission [51].

Weight loss has been observed as an adverse event in studies of tesofensine [19], prompting further research for the indication of obesity. The pharmaceutical company Neurosearch has conducted a phase IIb proof-of-concept dose-finding study and a subsequent study of metabolic outcomes using tesofensine; both of these studies indicate that tesofensine is efficacious in promoting weight loss in obese subjects [1]. The triple reuptake inhibitor sibutramine is approved by the United States Food and Drug Administration (FDA) for the indication of obesity. Research is generally lacking regarding the antidepressant potential of sibutramine, although a small study in obese and overweight subjects (N=60) suggests that it has mood-enhancing effects [25].

One published preclinical study describes the effect of the “balanced” triple reuptake inhibitor DOV 102,677 in reducing volitional alcohol consumption in ethanol-preferring rats without decreasing food or water consumption [28]. It should be noted that monoamine reuptake inhibitors have historically performed better in animal models of addiction than in human clinical trials. However, it is possible that agents which inhibit dopamine reuptake may offer improved efficacy in addictive disorders due to the link between dopamine and reward-motivated behaviors. Subsequent clinical trials in subjects with addictive disorders will elucidate the potential for triple reuptake inhibitors to reduce addictive behaviors.

## CONCLUSIONS

The impetus to develop triple reuptake inhibitors is a natural consequence of the rich drug development history occurring over the past fifty years. We have come a long way since the serendipitous discovery that TCAs and MAOIs exert antidepressant effects. Rational drug design has allowed us to customize the receptor profiles of potential antidepressant drugs and to target specific monoamine reuptake transporters. Current strategies involve developing multiple analogues of dual reuptake inhibitors and characterizing their receptor profiles in order to develop a quiver of molecules with clinically-relevant activity at all three monoamine reuptake sites. The ideal ratio of transporter site potencies that a triple reuptake inhibitor should exhibit remains unknown, and hopefully the diversity of molecules in development will shed light on this issue. Future research will undoubtedly involve clinical study of various triple reuptake inhibitors to determine whether any of them offer advantages over currently approved antidepressants in efficacy, rapidity of onset, or side effect profile. Research published to date tends to support that antidepressants vary modestly in various outcomes related to efficacy. Yet, findings are mixed regarding whether broader spectrum agents or highly serotonin-selective agents confer the best efficacy. Furthermore, concern has been expressed that triple reuptake inhibitors may produce broader side effect burden without enhancing efficacy over more selective agents [31]. In actuality, it is plausible that triple reuptake inhibitors that minimize blockade at histaminergic, cholinergic, and alpha-adrenergic receptors may yield the most favorable tolerability of all antidepressants with less sexual side effects than SSRIs or SNRIs. By way of example, bupropion has long been used to treat antidepressant-related sexual dysfunction, presumably through its dopaminergic effects [19]. Many of these questions will be answered by subsequent research.

## Figures and Tables

**Table 1:** Summary of Characteristics and Pharmacokinetic Parameters of Triple Reuptake Inhibitors in Development

Products	Compounds	Other Characteristics	Evidence of Antidepressant Potential	Half-Life (hr)	Tmax (Time to peak Concentration in hrs)	Affinity K_i_(nm/L) NE:SER:DA	Potency IC_50_(n M) NE:SER:DA
PRC025 [39]	(2*SR*, 3*RS*)-*N*,*N*-dimethyl-3-cyclohexyl-3-hydroxy-2-(2′-naphthyl)propylamine	Racemic analogue of venlafaxine	Animal/Preclinical			10: 6: 53	
PRC050 [39]	(2*RS*,3*RS*)-*N*-methyl-3-hydroxy-2-(2′-naphthyl)-3-phenylpropylamine	Racemic analogue of venlafaxine	Animal/Preclinical			1.2: 12: 43	
DOV 216,303 [4,44]	[(±)-1-(3,4-dichlorophenyl)-3-azabicyclo-[3.1.0]hexane hydrochloride].		Animal/Preclinical Small human clinical trial (not placebo-controlled)	Approx. 3.3 to 4.4	Approx. 1		21: 14: 78
DOV 21,947 [45]	[(+)-1-(3,4-dichlorophenyl)-3-azabicyclo-[3.1.0]hexane hydrochloride]	(+)-enantiomer of DOV 216,303	Animal/Preclinical			262: :99: 213	23: 12: 96
DOV 102,677 [34]	[(–)-1-(3,4-dichlorophenyl)-3-azabicyclo-[3.1.0]hexane hydrochloride])	(-)-enantiomer of DOV 216,303	Animal/Preclinical			1030: 740: 222	103: 133: 129
(-)-8h [37]	(-)-(1R, 2S)-naphthyl derivative of milnacipran		None				5: 18: 140
Bicifadine [3]	(+/-)-1-(4-methylphenyl)-3-azabicyclo-[3.1.0]hexane HCl)]	DOV 220,075	None	Approx. 3.5	Approx. 1	5.0: 2.4: 5.2 (µM)	55: 117: 910
Tesofensine (NS2330) [26]	8-azabicyclo[3.2.1]octane,3-(3,4-dichlorophenyl)-2-(ethoxymethyl)-8-methyl-, [1R-(2-endo,3-exo)]-,2-hydroxy-1,2,3-propanetricarboxylate		None	Approx. 230	Approx. 6-8		1.7: 11: 65

IC_50_: concentration required for 50% inhibition *in vitro*.

K_i_: binding affinity of the inhibitor.

NE:SE:DA = Norepinephrine: Serotonin: Dopamine.
